# Supplementary material for: Deletion of wfs1 Impairs Oligodendrocyte Precursor Cells Dorsal Distribution and Myelination Through the wfs1-hmgcs1 Axis in Zebrafish
Source: Biology (Basel). 2026 Aug 16;15(16):1402. doi: 10.3390/biology15161402 (PMC13510151; doi:10.3390/biology15161402)
Supplement: Supplementary file 1 [file biology-15-01402-s001.zip › File S1-Transcriptome sequencing data and differentially expressed genes (DEGs) in wfs1-- zebrafish at 5 dpf/2.KEGG/wfs1KOvsWT/GSEA/WT/HISTIDINE_METABOLISM/HISTIDINE_METABOLISM(DRE00340).html]

Details for gene set HISTIDINE\_METABOLISM(DRE00340)[GSEA]

|  || Dataset | deg.sample |
| Phenotype | sample.cls |
| Upregulated in class | WT |
| GeneSet | HISTIDINE\_METABOLISM(DRE00340) |
| Enrichment Score (ES) | -0.27948943 |
| Normalized Enrichment Score (NES) | -1.0255239 |
| Nominal p-value | 0.38247013 |
| FDR q-value | 0.5693985 |
| FWER p-Value | 1.0 |
Table: GSEA Results Summary

  

Fig 1: Enrichment plot: HISTIDINE\_METABOLISM(DRE00340)      
 Profile of the Running ES Score & Positions of GeneSet Members on the Rank Ordered List

  

| PROBE | DESCRIPTION (from dataset) | GENE SYMBOL | GENE\_TITLE | RANK IN GENE LIST | RANK METRIC SCORE | RUNNING ES | CORE ENRICHMENT || 1 | 368239 | aldh2.2 |  |  | 1221 | 1.881 | 0.0409 | No |
| 2 | 555401 | aoc1 |  |  | 1405 | 1.721 | 0.1078 | No |
| 3 | 447920 | aldh3a2b |  |  | 1830 | 1.444 | 0.1553 | No |
| 4 | 402826\_1 | si:ch73-71d17.1 |  |  | 1957 | 1.386 | 0.2098 | No |
| 5 | 558050 | si:ch211-127i16.2 |  |  | 4370 | 0.766 | 0.1664 | No |
| 6 | 100005587 | aldh9a1a.1 |  |  | 5134 | 0.663 | 0.1704 | No |
| 7 | 404730 | mao |  |  | 5766 | 0.610 | 0.1763 | No |
| 8 | 399481 | aldh9a1b |  |  | 6718 | 0.518 | 0.1683 | No |
| 9 | 393462 | aldh2.1 |  |  | 8615 | 0.394 | 0.1254 | No |
| 10 | 100000026 | aldh3a1 |  |  | 8825 | 0.379 | 0.1348 | No |
| 11 | 793609 | hdc |  |  | 9608 | 0.327 | 0.1240 | No |
| 12 | 492710 | aldh16a1 |  |  | 14167 | 0.084 | -0.0155 | No |
| 13 | 327288 | cndp2 |  |  | 20209 | -0.175 | -0.1977 | No |
| 14 | 394052 | ftcd |  |  | 20350 | -0.181 | -0.1945 | No |
| 15 | 282559 | aldh3b1 |  |  | 20742 | -0.200 | -0.1983 | No |
| 16 | 334197 | aldh7a1 |  |  | 21094 | -0.221 | -0.2000 | No |
| 17 | 100006238 | aldh9a1a.2 |  |  | 23161 | -0.338 | -0.2506 | No |
| 18 | 402826 | si:ch73-71d17.1 |  |  | 24027 | -0.394 | -0.2611 | Yes |
| 19 | 557008 | aldh3b2 |  |  | 24033 | -0.394 | -0.2447 | Yes |
| 20 | 556744 | uroc1 |  |  | 24709 | -0.431 | -0.2477 | Yes |
| 21 | 445242 | hnmt |  |  | 25723 | -0.505 | -0.2582 | Yes |
| 22 | 558050\_1 | si:ch211-127i16.2 |  |  | 26135 | -0.540 | -0.2483 | Yes |
| 23 | 557232 | aspa |  |  | 26403 | -0.567 | -0.2328 | Yes |
| 24 | 101886429 | LOC101886429 |  |  | 27507 | -0.674 | -0.2390 | Yes |
| 25 | 323653 | aldh3a2a |  |  | 28024 | -0.745 | -0.2238 | Yes |
| 26 | 503765 | carnmt1 |  |  | 28660 | -0.848 | -0.2080 | Yes |
| 27 | 100004331 | hal |  |  | 29573 | -1.085 | -0.1909 | Yes |
| 28 | 100006238\_1 | aldh9a1a.2 |  |  | 29971 | -1.231 | -0.1515 | Yes |
| 29 | 100005587\_1 | aldh9a1a.1 |  |  | 31308 | -2.447 | -0.0903 | Yes |
| 30 | 100536795 | wu:fb14d09 |  |  | 31372 | -2.574 | 0.0163 | Yes |
Table: GSEA details [plain text format]

  

Fig 2: HISTIDINE\_METABOLISM(DRE00340)      
 Blue-Pink O' Gram in the Space of the Analyzed GeneSet

  

Fig 3: HISTIDINE\_METABOLISM(DRE00340): Random ES distribution      
 Gene set null distribution of ES for **HISTIDINE\_METABOLISM(DRE00340)**
